# Toward improved thrombolysis in ischemic stroke: targeting nonfibrin components

**DOI:** 10.1016/j.rpth.2026.103434

**Published:** 2026-03-27

**Authors:** Simon F. De Meyer

**Affiliations:** Laboratory for Thrombosis Research, Department of Cardiovascular Sciences, KU Leuven Campus Kulak, Kortrijk, Belgium

**Keywords:** ischemic stroke thrombi, neutrophil extracellular traps, stroke, thrombolysis, von Willebrand factor

## Abstract

A State of the Art lecture titled “Targeting nonfibrin components in ischemic stroke” was presented at the International Society on Thrombosis and Haemostasis Congress in 2025. Fibrinolysis using recombinant tissue plasminogen activators remains the primary pharmacologic approach for reperfusion therapy in ischemic stroke. Its clinical benefit has been well established over the past 3 decades. However, fibrinolytic therapy is not universally effective, and its limitations have become increasingly apparent. Recent research has provided deeper insights into acute ischemic stroke thrombus composition and architecture, revealing complex structures containing not only fibrin but also cellular and extracellular components that contribute to fibrinolysis resistance. These findings have led to the development of novel strategies aimed at targeting nonfibrin components to enhance overall thrombolysis and overcome fibrinolysis resistance. This review highlighted the current evidence from studies on ischemic stroke thrombi that underpin emerging thrombolytic strategies that target nonfibrin components and discusses their potential clinical implications. Finally, relevant new data on this topic presented during the 2025 International Society on Thrombosis and Haemostasis Congress were summarized.

## 1 Ischemic Stroke

Acute ischemic stroke (AIS) is a major global cause of disability and death, placing a heavy burden on individuals and health care systems. It occurs when a thrombus blocks a cerebral artery, cutting off oxygenated blood to brain tissue and causing irreversible neuronal death in the infarct core. Surrounding this core is the ischemic penumbra, a region that can potentially be saved with timely reperfusion therapy to reduce long-term neurologic damage. The primary objective of acute reperfusion therapy is to achieve rapid recanalization of the occluded cerebral artery, either through pharmacologic dissolution (intravenous thrombolysis [IVT]) or mechanical removal (endovascular thrombectomy [EVT]) of the obstructing thrombus. IVT is based on fibrinolysis using recombinant tissue plasminogen activator (rtPA), a serine protease that facilitates thrombolysis by binding to fibrin within a thrombus and catalyzing the conversion of plasminogen to plasmin. The generated plasmin enzymatically degrades fibrin strands, leading to thrombus dissolution. Since its approval by the US Food and Drug Administration in 1996, rtPA (also known as alteplase) has become the standard first-line treatment for IVT. After nearly 3 decades of robust clinical evidence, rtPA remains the only currently approved fibrinolytic agent and remains the most widely implemented thrombolytic therapy worldwide. However, its use is constrained by several limitations. Besides several contraindications for its use, one of the primary challenges is its narrow therapeutic window, which restricts treatment to the limited time frame of 4.5 hours after symptom onset, resulting in <5% of patients globally receiving it in time [1]. Beyond this window, the risk-benefit profile becomes unfavorable, primarily due to an increased incidence of hemorrhagic complications. Notably, the fibrinolytic efficacy of rtPA is relatively modest, with arterial recanalization achieved in <50% of cases, limiting its effectiveness in restoring cerebral blood flow [[1], [2], [3]]. The mechanisms underlying resistance to rtPA are not fully understood but may include large thrombus size, delayed initiation of treatment, collateral status, and specific thrombus composition, as will be discussed further. These limitations have led to growing interest in alternative fibrinolytic agents such as tenecteplase, a genetically modified form of tissue plasminogen activator (tPA) with a longer half-life, greater fibrin specificity, and reduced inactivation by plasminogen activator inhibitor 1 (PAI-1). Notably, its simplified single-bolus administration makes tenecteplase a promising alternative, and ongoing clinical trials are evaluating its efficacy and safety compared to alteplase [4].

Besides IVT, EVT has significantly transformed acute reperfusion treatment for ischemic stroke over the past decade, particularly in cases involving large vessel occlusions (LVOs). EVT has demonstrated substantial clinical benefits due to its ability to achieve rapid and effective reperfusion. Nevertheless, EVT is restricted to a relatively small subset of stroke patients—specifically those presenting with LVOs and who have timely access to specialized thrombectomy centers. Consequently, IVT remains a widely used reperfusion strategy on a global scale. Considering the inherent limitations of current IVT protocols and the stringent safety measures required for their administration, there is an urgent need to develop next-generation thrombolytic agents that demonstrate enhanced efficacy while offering a more favorable risk-benefit profile.

## 2 Insights from Studying Ischemic Stroke Thrombus Composition

Although the occluding thrombus is the primary target of reperfusion therapies such as IVT and EVT, its detailed composition and structural characteristics have remained poorly understood. This knowledge gap has persisted largely due to the inaccessibility of thrombi for direct analysis. However, the advent of EVT has enabled the retrieval of thrombotic material during stroke intervention, facilitating direct access to patient-derived thrombi. This breakthrough has opened valuable opportunities for systematic investigation into thrombus biology, marking the emergence of a specialized subfield in stroke research focused on the histologic and structural analysis of AIS thrombi and their impact on treatment efficacy [5]. It has become clear that thrombi retrieved from different stroke patients exhibit substantial heterogeneity in both composition and architecture [6]. These clots display complex structural profiles, comprising varying proportions of thrombotic and inflammatory components such as red blood cells (RBCs), platelets, von Willebrand factor (VWF), fibrin, leukocytes, and neutrophil extracellular traps (NETs). This diversity highlights the intricate nature of ischemic stroke thrombus biology and suggests potential implications for therapeutic approaches such as IVT and EVT [7]. The mechanisms underlying thrombus organization and compositional heterogeneity remain incompletely understood, but factors such as local hemodynamics, thrombus age, maturation, and contraction likely contribute to the observed variability.

**Figure 1:**
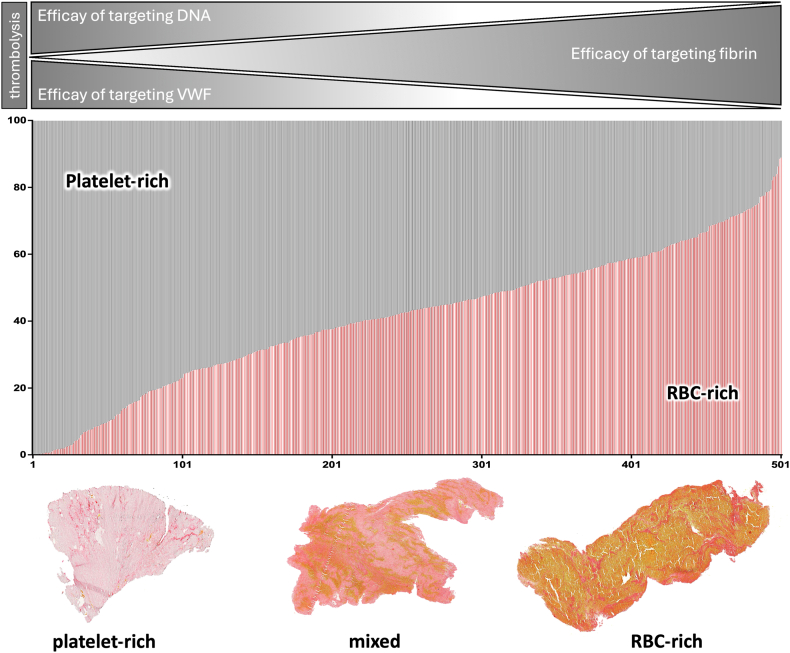
Heterogeneity in the composition and thrombolysis susceptibility of acute ischemic stroke thrombi. Thrombi retrieved from patients with acute ischemic stroke (*n* = 501; represented as vertical bars) were analyzed using histologic staining to determine the relative proportions of platelet-rich (grey) and red blood cell (RBC)-rich (red) material. The composition of stroke thrombi varies widely, ranging from predominantly platelet-rich (left) through mixed composition (middle) to thrombi primarily composed of RBC-rich material (right). Representative images of a platelet-rich thrombus (left), a mixed thrombus (middle), and an RBC-rich thrombus (right) are shown below the graph. These samples were stained using Martius Scarlet Blue, which colors RBCs yellow and fibrin dark pink/red. The thrombolysis efficacy targeting fibrin and nonfibrin components is shown above the graph. Accumulating evidence indicates that the efficacy of fibrinolysis with plasminogen activators (PAs) correlates with thrombus composition, with fibrinolytic efficacy decreasing as RBC content declines. Conversely, strategies targeting nonfibrin components such as DNA and von Willebrand factor in combination with recombinant tissue PA (rtPA) appear particularly effective in platelet-rich thrombi, while offering limited additional benefit in RBC-rich thrombi that are already susceptible to rtPA. *Source:* Figure adapted from Staessens et al. [44], with permission.

**Figure 2:**
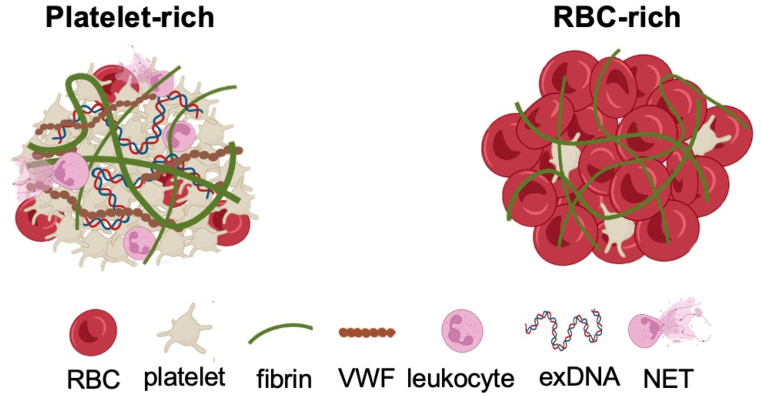
Structural composition of platelet-rich and red blood cell (RBC)-rich thrombi in acute ischemic stroke. Schematic representation of a platelet-rich thrombus (left) and an RBC-rich thrombus (right). RBC-rich thrombi exhibit a relatively simple architecture composed primarily of RBCs interspersed with fibrin fibers. In contrast, platelet-rich thrombi display a more complex structure, containing not only platelets and fibrin but also von Willebrand factor (VWF), leukocytes, extracellular DNA (exDNA), and neutrophil extracellular traps (NETs). *Source:* Figure taken from Vandelanotte and De Meyer [92], with permission.

Through a systematic analysis of thrombi retrieved from patients with AIS, Staessens et al. [8] delineated 2 distinct organizational categories of ischemic stroke thrombi—RBC-rich and platelet-rich—based on their molecular and cellular composition. Most thrombi retrieved from patients exhibit a heterogeneous mixture of these 2 types, with considerable variation in their relative proportions (Figure 1). RBC-rich regions are characterized by a relatively simple structure of densely packed RBCs embedded in a thin fibrin network, with uniformly distributed leukocytes. In contrast, platelet-rich areas display a more complex architecture, comprising dense fibrin fibers, VWF, NETs, and platelet aggregates, with leukocytes and extracellular DNA often concentrated at the interface between RBC-rich and platelet-rich zones (Figure 2).

Accumulating evidence indicates that the cellular composition of thrombi critically influences their susceptibility to rtPA. Thrombi with high RBC content demonstrate greater responsiveness to rtPA-mediated fibrinolysis compared with those predominantly composed of platelets, which tend to exhibit increased resistance to fibrinolytic therapy (Figure 1) [[9], [10], [11], [12], [13], [14]]. Using thrombi retrieved from patients with AIS, Vandelanotte et al. [14] recently demonstrated the efficacy of rtPA-mediated thrombolysis is strongly correlated with the composition. Specifically, thrombi with a high RBC content were efficiently degraded by rtPA, whereas those with low RBC content exhibited marked resistance to fibrinolysis [14]. The relatively simple architecture dominated by a fibrin scaffold most likely renders RBC-rich thrombi largely susceptible to plasmin-mediated fibrinolysis. In contrast, the more complex structure of platelet-rich thrombi, including also nonfibrin components such as VWF, extracellular DNA, and NETs, may impair fibrinolytic efficacy. Below, the key structural components and their potential modulatory effect on thrombolysis are discussed.

### 2.1 Fibrin

The final step of the coagulation cascade is the thrombin-mediated conversion of fibrinogen into insoluble fibrin, which polymerizes to form an interconnected network that stabilizes the developing thrombus. As the principal substrate targeted by plasminogen activators such as alteplase and tenecteplase during fibrinolytic therapy, the abundance, structural organization, and accessibility of fibrin are critical determinants of therapeutic efficacy. Given the focus of this review on nonfibrin components, readers are referred to the excellent literature detailing the general structure and properties of fibrinogen and fibrin and their role in clot architecture [[15], [16], [17], [18]]. Interestingly, in pathologic settings, including AIS, fibrin clot properties can be unfavorably altered, including faster formation of denser and poorly lysable fiber networks [19]. In thrombi collected from patients with AIS, the abundance and structural organization of fibrin vary considerably among stroke thrombi. In RBC-rich regions, fibrin forms a thin, branched network, whereas in platelet-rich areas, it appears as dense bundles that delineate platelet clusters [8]. Di Meglio et al. [20] have revealed that fibrin can form a continuous, dense outer shell around the thrombus that impairs fibrinolysis. Besides tightly packed fibrin, this shell also contains VWF, aggregated platelets, and platelet-derived antifibrinolytic components such as PAI-1 and protease nexin-1. Such a thrombus shell resembles previously described fibrin films in which fibrin molecules align into continuous layers that form a protective surface coating, a structural feature reported in thrombi across diverse cardiovascular settings [21]. Additional chemical modifications of fibrin can further enhance its resistance to fibrinolysis, including FXIII-mediated crosslinking of antifibrinolytic proteins such as α2 antiplasmin [22] and PAI-2 [23], as well as glycosylation [24], oxidation [25], and carbamylation [26]. Notably, the prevalence, regulation, and functional impact of these modifications remain incompletely characterized in the specific setting of ischemic stroke, highlighting an interesting area for future research.

### 2.2 Platelets

Platelets are central to thrombus formation. Following vascular injury, platelets adhere directly to exposed subendothelial collagen via glycoprotein VI and integrin α2β1, or indirectly through VWF binding to the glycoprotein Ib-V-IX complex. Platelet activation induces a conformational change in integrin αIIbβ3, enabling fibrinogen- and VWF-mediated aggregation. The subsequent release of platelet granule contents, including VWF, fibrinogen, ADP, and thromboxane A_2_, amplifies platelet adhesion, activation, and aggregation. Consistent with this role, AIS thrombi contain prominent platelet-rich regions characterized by dense fibrin and VWF networks forming platelet islands [8]. Within AIS thrombi, the presence of platelets can contribute to resistance to fibrinolysis through multiple mechanisms. Platelet-derived polyphosphates modulate fibrin fiber thickness and branching, promoting the formation of a denser and more compact fibrin network [27,28]. In addition, platelets store and release key antifibrinolytic factors, including PAI-1 and thrombin-activatable fibrinolysis inhibitor, which suppress rtPA-mediated plasminogen activation. Both inhibitors have been detected at high levels within platelet-rich, rtPA-resistant regions of AIS thrombi. [20,29]. Importantly, platelets are known to mediate clot contraction [30,31]. Clot contraction is mediated by cytoskeletal motility within activated platelets, which generate tensile forces that are transmitted through platelet filopodia and fibrin via integrin αIIbβ3, resulting in clot compaction, serum expulsion, and the spatial redistribution of cellular and protein constituents [30]. This process may at least partially account for the spatial organization observed in AIS thrombi, characterized by densely packed erythrocyte-rich regions interspersed with fibrin- and platelet-rich domains. By reducing clot porosity and limiting fibrinolytic enzyme infiltration, platelet-mediated clot contraction represents an additional mechanism contributing to resistance to rtPA-mediated clot lysis [32].

### 2.3 RBCs

RBCs are almost universally present in AIS thrombi, yet their abundance and distribution vary widely, ranging from RBC-rich clots to those nearly devoid of them (Figure 1). RBCs are well-known to regulate clot structure and thrombosis [33]. Within ischemic stroke thrombi, RBCs are typically embedded among thin fibrin strands, and RBC-dense regions often alternate with platelet-rich areas. Clot contraction deforms RBCs from their normal biconcave shape into tightly packed polyhedral structures known as polyhedrocytes [30,34], which are a common morphologic RBC feature found in stroke thrombi [[35], [36], [37], [38]]. Clot contraction was reported to be less potent in AIS plasma clots [39], but platelet-mediated clot contraction and RBC compaction can still result in compressed, dense thrombi that are more impermeable, negatively affecting thrombolysis [30,38,40,41]. On the other hand, however, increased RBC content within a thrombus has also been associated with looser fibrin networks with enlarged pore sizes, facilitating penetration of thrombolytic agents, potentially enhancing the efficiency of clot dissolution [42,43]. Together, these observations highlight the heterogeneous roles of RBCs in AIS thrombi and their modulatory influence on thrombolysis, underscoring the need for further investigation into the mechanisms by which RBCs contribute to variability in thrombolytic efficacy.

### 2.4 von Willebrand factor

Alongside fibrin, VWF is a major noncellular component of stroke thrombi. VWF is a large multimeric protein essential for platelet adhesion and thrombus formation, released by endothelial cells and platelets during vascular injury, inflammation, or ischemia. Upon exposure to subendothelial collagen or high shear stress, VWF binds to platelet glycoprotein Ibα, initiating platelet adhesion and aggregation. It also acts as a molecular bridge between activated platelets, contributing to thrombus stabilization. Immunohistochemical studies have consistently demonstrated the presence of VWF in AIS thrombi, particularly in platelet-rich regions, where its abundance can range from 1% to over 90%, and in the shell [8,[44], [45], [46], [47], [48]]. VWF frequently colocalizes with fibrin in dense structures that align platelet aggregates [8]. This close interaction may result from covalent binding via factor XIII, potentially playing a key role in thrombus stabilization [49]. These findings suggest that, similar to fibrin, VWF contributes to a structural framework within AIS thrombi, potentially enhancing its stability and reducing its susceptibility to rtPA-mediated fibrinolysis.

### 2.5 Neutrophil extracellular traps

In addition to fibrin and VWF, NETs have emerged as key structural components of thrombi in AIS [50,51]. Initially described as a novel immune defense mechanism, NETs are formed when neutrophils release decondensed chromatin decorated with antimicrobial proteins, creating a meshwork of extracellular DNA fibers [52]. Beyond their immune function, NETs contribute to thrombogenesis by serving as a scaffold for thrombus formation in both arterial and venous contexts [53,54]. Multiple studies have confirmed the presence of NETs in stroke thrombi through immunostaining of neutrophil markers (eg, CD66b, neutrophil elastase, and myeloperoxidase) alongside NET-specific markers such as citrullinated histones and extracellular DNA [8,50,51,[55], [56], [57], [58]]. NETs predominantly accumulate in platelet-rich regions and at the interface between platelet-rich and RBC-rich zones [8]. Using Feulgen’s staining, a method highly sensitive to all DNA, and thus not limited to NETs, extensive DNA networks have been observed in these regions [8]. While the exact origin of these networks remains uncertain, they may derive from NETs, circulating DNA, or necrotic cells. It has become evident that extracellular DNA networks also contribute to rtpA resistance [50,51,59]. While extracellular DNA networks and NETs form structural scaffolds that are intrinsically resistant to fibrinolytic degradation, they can further impair therapeutic thrombolysis through several mechanisms. These include the accumulation of antifibrinolytic factors, including PAI-1 and α_2_-antiplasmin [60,61]; sequestration of key fibrinolytic molecules, including tPA and plasminogen, diverting them away from fibrin [61]; promotion of platelet-driven thrombus contraction [62]; and modulation of fibrin structure toward thicker fibers and denser thrombi [[63], [64], [65]].

## 3 Targeting Nonfibrin Components as Adjunct Thrombolytic Strategies

Given the structural complexity and compositional heterogeneity of thrombi, it is increasingly evident that a uniform “one-size-fits-all” approach to thrombolytic therapy—such as the use of rtPA alone—is unlikely to be universally effective across all thrombus types. Therefore, therapeutic strategies that move beyond fibrin-centered thrombolysis and target nonfibrin structural components represent a promising avenue to enhance thrombolytic efficacy, particularly in rtPA-resistant, platelet-rich thrombi. This section highlighted emerging approaches aimed at dismantling 2 major nonfibrin scaffolds, VWF and extracellular DNA that are highly enriched in platelet-rich thrombi (Figure 1).

### 3.1 DNA

NETs, and extracellular DNA more broadly, have emerged as prominent nonfibrin targets in efforts to enhance the efficacy of thrombolytic therapy. The DNA-cleaving enzyme DNase-1 degrades extracellular DNA, thereby disrupting the structural integrity of the DNA matrix within thrombi. Leveraging this principle to enhance thrombolysis, we and others have demonstrated in *ex vivo* experiments using human AIS thrombi that combining rtPA with recombinant DNase-1 significantly improves thrombus dissolution [14,50,51,61,66,67]. Our recent data demonstrate that the combination of DNase-1 with rtPA is particularly effective in thrombi with a high platelet content, which aligns with the enrichment of NETs in these thrombi [14]. Beyond disrupting the DNA scaffold that may contribute to thrombus structural integrity, the thrombolytic benefit of degrading extracellular DNA is also attributable to the potentiation of rtPA-mediated fibrinolysis, most likely through degradation of NETs, which act as decoy binding sites for tPA, titrating it away from fibrin and lifting NET-related antifibrinolytic activities [61,68]. The clinical variant of DNase-1, known as Dornase alfa (Pulmozyme), has entered clinical trials to improve reperfusion rates (EXTEND-IA DNase, NCT05203224, and NETs-target, NCT04785066) and reduce stroke-related systemic inflammation (ReSCInD, NCT058805524).

### 3.2 von Willebrand factor

The abundance of VWF in thrombi from patients with AIS strengthened the rationale for targeting VWF as a novel approach to thrombolysis. Emerging therapeutic strategies focus on either degrading large VWF multimers or inhibiting their interaction with platelets to disrupt thrombus stability and enhance reperfusion.

ADAMTS-13 (a disintegrin and metalloproteinase with a thrombospondin type 1 motif, member 13) is a metalloprotease that cleaves ultralarge VWF multimers at the Tyr1605-Met1606 bond within the A2 domain, reducing their size and adhesive potential. By dismantling these highly prothrombotic multimers, ADAMTS-13 can destabilize platelet-rich thrombi, making it an attractive mechanism for thrombolysis in ischemic stroke. To evaluate its thrombolytic efficacy, we induced occlusive VWF-rich thrombi in the middle cerebral artery of mice. While rtPA failed to resolve these occlusions, recombinant ADAMTS-13 promoted dose-dependent dissolution of rtPA-resistant thrombi, leading to rapid restoration of arterial patency and significant reduction in cerebral infarct size [45]. These results were further corroborated using structural mutants of ADAMTS-13, demonstrating good thrombolytic efficacy in platelet-rich rtPA-resistant thrombi [[69], [70], [71]]. The recent approval of recombinant ADAMTS-13 for congenital thrombotic thrombocytopenic purpura provides a foundation for exploring its therapeutic relevance in cerebrovascular disorders such as stroke [72]. Notably, release of peptidylarginine deiminase type IV during NETosis has been shown to induce citrullination and functional inactivation of ADAMTS-13, thereby impairing VWF cleavage [73]. Whether this regulatory mechanism is operative in the context of ischemic stroke remains unclear. Addressing this question is particularly relevant, as protein arginine deiminase 4 inhibition could potentially potentiate ADAMTS-13 activity and thereby enhance ADAMTS-13-mediated thrombolysis in NET-rich, platelet-rich thrombi.

N-acetylcysteine (NAC) is a US Food and Drug Administration-approved drug that has long been used to treat chronic obstructive pulmonary disease and acetaminophen toxicity, where it functions by reducing mucin multimers. Chen et al. [74] demonstrated that NAC can similarly reduce VWF multimers by cleaving disulfide bonds between VWF monomers, whose polymerization resembles that of mucins, leading to rapid thrombus resolution in ADAMTS-13–deficient mice. Preclinical studies, including thromboembolic stroke models in mice and analyses of thrombi retrieved from patients with stroke, have shown that NAC and its derivative N,N'-diacetyl-L-cystine can effectively dissolve VWF-rich thrombi, suggesting their potential to enhance thrombolysis, particularly in cases resistant to rtPA [14,[75], [76], [77], [78]]. A randomized, open-label pilot study evaluated the safety and efficacy of NAC as an adjunct to alteplase in patients with AIS treated within 4.5 hours of onset [79]. While no significant differences were found in safety outcomes or 3-month mortality between the NAC + rtPA and rtPA-only groups, the NAC-treated group showed a statistically significant improvement in early neurologic recovery (National Institutes of Health Stroke Scale at 24 hours) while long-term functional outcomes at 3 months remained comparable [79]. Recently, however, a single-group clinical trial of intravenous NAC as an adjunct thrombolytic therapy to alteplase was terminated early despite demonstrating a significant reduction of circulating ultralarge VWF multimers due to the occurrence of 2 fatal intracranial hemorrhages in patients with prior antiplatelet therapy, underscoring the need for larger trials to further assess the safety and therapeutic potential of NAC in stroke [80].

It has been proposed that plasminogen activation can act as a natural backup for ADAMTS-13 to degrade obstructive platelet-VWF complexes [81]. Building on this concept, Microlyse (TGD-001) has been developed as a single polypeptide consisting of a VWF-targeting nanobody and the catalytic domain of urokinase plasminogen activator for plasmin-mediated destruction of platelet-VWF complexes. Microlyse binds with high affinity to the VWF C-terminal cystine knot domain, initiating localized plasminogen activation, triggering plasmin-mediated degradation of VWF multimers [82]. Based on the idea that plasmin can degrade both fibrin and VWF, the efficacy of Microlyse and rtPA was tested in both a fibrin- and a platelet-rich mouse model of ischemic stroke [83]. In the fibrin-rich model, both rtPA and Microlyse enhanced cortical reperfusion and reduced cerebral lesion volume. In contrast, in the platelet-rich model, neither agent improved reperfusion at 10 minutes posttreatment; however, Microlyse, unlike rtPA, significantly decreased lesion volume. These results indicate that Microlyse may offer broad therapeutic potential in ischemic stroke, regardless of thrombus composition [83]. A phase 1a clinical trial, assessing TGD001’s safety and tolerability in healthy volunteers, was recently announced (European Union Clinical Trial Identification Number: 2024-514931-63-00).

Aptamers are a class of DNA or RNA ligands that fold into 3-dimensional structures, binding to and inhibiting the function of their target proteins with high affinity and specificity. BB-031 is an RNA aptamer that targets VWF and, together with its complementary reversal agent BB-025, enables controlled inhibition by specifically blocking the VWF A1 domain responsible for platelet binding [84]. In a canine model of carotid and basilar artery occlusion, where autologous clots were placed into the basilar artery to induce stroke, BB-031 provided superior recanalization of the basilar artery and surrounding territory, resulting in significantly smaller infarct volumes compared to vehicle and the clinical standard rtPA [85]. Phase 1 clinical trials established safety in healthy volunteers, and a phase 2 clinical trial is currently ongoing.

## 4 International Society on Thrombosis and Haemostasis Congress Report

At the 2025 International Society on Thrombosis and Haemostasis Congress, several noteworthy abstracts were presented on this topic. Solo Nomenjanahary et al. reported an intriguing study demonstrating how DNase I can unlock the thrombolytic potential of intravenously administered tPA in ischemic stroke thrombi [86]. The researchers aimed to determine whether biologically relevant intrathrombus concentrations of tPA are achieved following IVT in patients with stroke with LVOs and whether extracellular DNA contributes to IVT failure. To address these questions, 136 thrombi from patients with AIS with LVOs were analyzed, 69 treated with IVT, and 67 without. Of these, 83 were assessed for tPA content, and 53 underwent *ex vivo* thrombolysis with plasminogen, with or without DNase I. Interestingly, thrombi from the IVT group contained significantly more tPA, which was associated with both fibrin and NETs. Plasminogen alone did not induce thrombus lysis, but combined with DNase I, it significantly reduced thrombus weight, especially in IVT samples. DNase I also promoted fibrinolysis and released plasmin inhibitors, suggesting that extracellular DNA retains fibrinolysis inhibitors. These findings indicate that intrathrombus tPA concentrations achieved after IVT have therapeutic potential, but this effect is impaired by extracellular DNA, which may be unlocked by DNase I [72].

Emily Mihalko presented a study investigating the recanalization efficacy of BB-031 in microfluidic thrombotic occlusions using blood samples from healthy individuals and patients with ischemic stroke [87]. In an *in vitro* microfluidic platform, heparinized blood from either healthy donors or patients with stroke was perfused through a collagen-coated, stenosed chamber mimicking arterial occlusion. Formed thrombi exhibited a high platelet content and completely occluded the channel. Treatment with BB-031 significantly increased outflow compared with controls, alteplase, and tenecteplase, demonstrating the recanalization efficacy of BB-031 and warranting its further development as a therapeutic adjunct or alternative to current standards of care for ischemic stroke [73].

Rebecca Risman shared interesting findings on fibrin rearrangements during clot contraction and their potential role in accelerating internal fibrinolysis [88]. While it is well established that platelet-driven clot contraction limits external fibrinolysis (the exogenous delivery of fibrinolytic agents), less is known about the mechanisms that enhance internal fibrinolysis (the physiological degradation of the fibrin network) within contracted clots [89,90]. By examining clot contraction and fibrinolysis in clots formed from platelet-rich plasma and platelet-poor plasma, the authors demonstrated that both platelet-driven tension and clot volume shrinkage were needed for clot contraction and accelerated internal fibrinolysis. Clot contraction led to the formation of a dense fibrin periphery with reduced pore size and a heterogeneous fibrin network in the core, resulting from platelet-driven fibrin fiber rearrangement. The authors proposed that accelerated internal fibrinolysis arises from the overall looser, rearranged fibrin network within the core and the eventual dismantling of the dense peripheral fibrin layer [74]. These findings underscore the need for future studies examining how platelet-mediated, contraction-induced rearrangements within the clot core and periphery affect thrombolytic strategies targeting nonfibrin components. For example, Schartz et al. [91] reported that ADAMTS-13–enhanced rtPA-mediated thrombolysis is particularly pronounced in clots exhibiting higher computed tomography-perviousness (an imaging feature commonly used as a surrogate for clot porosity or permeability), which were characterized by an increased proportion of platelet-associated proteins. Further investigation is warranted to elucidate how contraction-driven remodeling of clot regions affects porosity, fibrinolysis, and the efficacy of adjunctive thrombolytic approaches.

## 5 Future Directions

Advances in AIS reperfusion therapy have broadened treatment options, with EVT and alternative thrombolytics offering improved outcomes for patients. Future research may further focus on strategies targeting nonfibrin components such as DNA structures and VWF multimers. A deeper understanding of the underlying mechanisms underlying their beneficial thrombolytic effect will be crucial to fully realize their therapeutic potential. Promising avenues include the development of combination strategies—“thrombolytic cocktails”—that integrate agents like DNase, ADAMTS-13, NAC, and VWF-targeted fusion proteins, alongside further optimization of rtPA molecules and administration protocols to maximize efficacy. Rigorous evaluation of safety profiles, dosing regimens, routes, and timing of administration and translational pathways through well-designed clinical trials will be essential to bring these innovations into practice. Together, such new insights may ultimately guide optimized treatment strategies and enhance clinical outcomes in ischemic stroke [92].
